# Effects of two‐dose intravenous administration of furosemide on clinical variables, electrocardiographic indices and serum electrolytes in dromedary calves

**DOI:** 10.1002/vms3.365

**Published:** 2020-09-23

**Authors:** Amir Saeed Samimi, Ali Sanjarinejad

**Affiliations:** ^1^ Department of Clinical Science Faculty of Veterinary Medicine Shahid Bahonar University of Kerman Kerman Iran

**Keywords:** dromedary calves (*Camelus dromedarius*), electrocardiographic indices, furosemide, low and high dose, macrominerals

## Abstract

The intravenous (IV) usage of diuretic agents such as furosemide may cause changes in clinical signs, electrocardiographic (ECG) indices and serum electrolytes (sodium, potassium, chloride, calcium, phosphorus and magnesium) concentrations in dromedary calves. The purpose of this study was to evaluate the clinical, ECG and biochemical effects of two‐dose IV administration of furosemide in dromedary calves. A total of 21 clinically healthy male dromedary calves with the age of 5 (± 1) months and weight of 95 (± 5) kg were studied. The animals were randomly divided into three groups of control (normal saline), low‐dose furosemide (2.5 mg/kg) and high‐dose furosemide (5 mg/kg). Two IV injections with 12‐hr intervals were administered in all animals. The clinical and ECG parameters were measured at 0 (baseline), 2 (T2), 24 (T24) and 48 (T48) hours after drug administration. Serum concentrations of electrolytes were measured at T0, T24 and T48 hr. The results of this study showed no changes in clinical parameters (heart rate, rectal temperature, respiratory rate and Unruminal motility), ECG indices and also no significant changes in serum electrolytes levels. Under conditions with free access to fresh water, two‐dose IV injection of furosemide (2.5 and 5 mg/kg) could be administered in healthy dromedary calves.

## Graphical abstract

The results of this study showed no changes in clinical parameters (heart rate, rectal temperature, respiratory rate and ruminal motility), ECG indices and also no significant changes in serum electrolytes levels. Two‐dose IV injection of furosemide (2.5 and 5 mg/kg) could be administered in healthy dromedary calves.

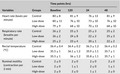

## 1 INTRODUCTION

Diuretic are prescribed to excrete additional extracellular fluids by increasing urine flow, sodium excretion and decreasing hypertension. There is a need to use these drugs in many veterinary treatments, such as oedema and congestive heart failure, udder and pulmonary oedema, ascites and acidosis or alkalosis, as well as dilution and faster excretion of toxins, especially those that are effective on the renal glumerular filtration rate (Pourjafar et al., 2013; Raidal et al., 2014). The function of the current diuretics include: loop, osmotic, potassium retainers and carbonic anhydrate inhibitors. The loop diuretics are very powerful drugs that prevent tubular reabsorption of sodium, chloride and other electrolytes from kidney tubules, increase their excretion and urine flow. Furosemide is considered to be one of the widely used drugs while also having some side effects, particularly serum potassium and sodium depletion (Ali et al., 1997; Gharaibeh, 1999; Yildiz & Ok, 2017).

There were limited information on the effect of two‐dose intravenous (IV) administration of furosemide on clinical variables, electrocardiographic (ECG) indices and serum electrolytes levels in camelide, especially calf‐camels (Ali et al., 1998; Chalmeh & Mazrouei Sebdani, 2016; Santamarina et al., 2001). The purpose of this study was to investigate the effects of two‐dose IV administration of furosemide on clinical variables, ECG indices and serum electrolytes levels in the dromedary calves.

## 2 MATERIALS AND METHODS

### 2.1 Animals

The present study was carried out in the Faculty of Veterinary Medicine, Shahid Bahonar University of Kerman, Kerman, southeast of Iran. Dromedary calves were selected from the Large Animal Research Center of the Faculty of Veterinary Medicine, Shahid Bahonar University of Kerman. The calves had been domesticated and information (including sex, date of birth and history of vaccination and health status) of the calves recorded precisely.

These animals were kept under the same management conditions and received a diet close to the maintenance and physiological requirements as set by Laudadio et al. (2009). Roughage (containing corn silage and alfalfa hay) supplemented with minerals (0.3%) was considered for diet of animals. At all stages of the study, the feed was distributed ad libitum in two meals at 9 a.m. and 6 p.m. Two months before the start of the experiment, all animals were treated with broad‐spectrum antiparasitic agents (by administration of oral albendazole, 15 mg/kg, and subcutaneous ivermectin, 0.2 mg/kg) to control possible contamination of internal and external parasites. At the baseline, the clinical and laboratory status of the animals was evaluated and only clinically and paraclinically healthy animals were allocated in the study. The experiment was carried out in the morning. The ambient temperature and relative humidity during the experiments were 19°C–21°C and 12%–14%, respectively. The experiments were carried out in an outdoor covered 6 × 7 m area. The animals were weighed before each treatment for calculation of drug dosages. The calves were moved to the experiment area and restrained in a quiet sternal position on a soft and comfortable mattress. Three or four calves were studied at any one time. Skin over the left jugular vein was clipped and scrubbed with povidone‐iodine for IV administration and blood sampling.

### 2.2 Experimental procedures

The current study was conducted on 21 clinically healthy male dromedary calves (*Camelus dromedarius*) of age 5 (± 1) months and weight of 90 (± 5) kg. The animals were randomly divided into three groups (seven animals in each group) as follows: control (normal saline), low‐dose furosemide (2.5 mg/kg; Vetazomide^®^, Aburaihan Pharma Co) and high‐dose furosemide (5 mg/kg). Two IV injections with 12‐hr interval were administered in all animals. The clinical parameters (including heart rate (HR), respiratory rate (RR), rectal temperature (RT) and ruminal motility) and ECG indices were measured at 0 (baseline), 2 (T2), 24 (T24) and 48 (T48) hours after drug administration. Serum concentrations of different electrolytes were measured at T0, T24 and T48.

### 2.3 Clinical findings and ECG indices

HR was evaluated using stethoscope (Classic II SE, Littmann Co) on the left side of chest wall (fourth intercostal space, behind the olecranon), and RR was assessed using direct observation of thoraco‐abdominal movement for 1 min. RT was measured using a digital thermometer inserted into the rectum. Ruminal motility (number of audible rumen contraction within 2‐min auscultation) was assessed using stethoscope placed on the left paralumbar fossa. Two large animal internists who were unaware of the drug evaluated the clinical parameters. ECG was obtained from all animals by bipolar base‐apex lead method similar to the method of Samimi et al. (2020).

### 2.4 Biochemical parameters

Blood samples were taken from all animals via jugular vein and kept in test tube with no anticoagulant. For blood biochemistry, samples were kept at 24ºC for 1 hr and then centrifuged (10 min, 3,000 g). Sera were separated and stored at −20°C for 1 day (before variables measurement). Sera were analysed for sodium and potassium values by flame photometry (FLM, Ontario, Canada). Serum concentrations of chloride and phosphorus were measured using UV spectrophotometer. Atomic absorption spectrophotometry (Shimadzu AA‐670) was used to determine calcium and magnesium levels.

### 2.5 Statistical analysis

Data (clinical variables, ECG indices and serum electrolytes levels) were expressed as mean ± standard deviation (*SD*). Data normality was analysed by Kolmogorov–Smirnov test before statistical analysis. Data obtained from the same time between different groups were analysed using one‐way analysis of variance (ANOVA). Repeated measures ANOVA were applied to compare the quantitative data during different times in each group. Also, paired sample *t* test was used to compare the mean values of quantitative data at different time points with baseline in each group. SPSS version 23 software (SPSS for Windows, SPSS Inc.) was used for all statistical analyses. Statistically significance level was considered to be *p* < .05.

## 3 RESULTS

Changes in clinical variables and ECG indices are shown in Tables 1 and 2, respectively. There was no statistically significant difference in HR, RR, ruminal motility, RT and ECG indices among different groups at all time points. Changes in electrolytes concentrations in different groups are shown in Table 3. There were no significant differences in serum electrolytes concentrations among different groups at any time point. All parameters were not significant changes from baseline in all groups after drug administration.

**TABLE 1:** Mean (± standard deviation) clinical variables immediately before (baseline) and up to 48 hr following two‐dose intravenous administration of low dose (2.5 mg/kg) and high dose (5 mg/kg) of furosemide, and normal saline (control) in dromedary calves (*Camelus dromedarius*)

Variables	Groups	Time points (hrs)			
		Baseline	120	24	48
Heart rate (beats per minute)	Control	80 ± 8	81 ± 9	76 ± 13	81 ± 9
	Low dose	80 ± 11	76 ± 10	73 ± 10	76 ± 10
	High dose	79 ± 9	68 ± 10	70 ± 13	68 ± 10
Respiratory rate (breaths per minute)	Control	26 ± 2	25 ± 5	25 ± 3	25 ± 2
	Low dose	26 ± 2	24 ± 8	22 ± 2	25 ± 3
	High dose	25 ± 2	23 ± 4	20 ± 1	22 ± 2
Rectal temperature (°C)	Control	36.4 ± 0.4	36.4 ± 0.2	36.3 ± 1.2	36.4 ± 0.2
	Low dose	35.5 ± 1	36.1 ± 1.2	35.9 ± 1.1	35.7 ± 1
	High dose	35.5 ± 1	36 ± 1.2	35.8 ± 1.1	35.7 ± 1
Ruminal motility (contraction per 2 min)	Control	2 ± 0	2 ± 0	2 ± 0	2 ± 0
	Low dose	2 ± 0	1 ± 1	2 ± 0	1 ± 1
	High dose	2 ± 0	2 ± 0	1 ± 1	2 ± 0

**TABLE 2:** Mean (± standard deviation) electrocardiographic indices immediately before (baseline) and up to 48 hr following two‐dose intravenous administration of low dose (2.5 mg/kg) and high dose (5 mg/kg) of furosemide, and normal saline (control) in dromedary calves (*Camelus dromedarius*)

ECG indices		Groups	Time points (hrs)			
			Baseline	120	24	48
Amplitude (mv)	*p*	Control	0.1 ± 0.01	0.1 ± 0.01	0.09 ± 0.01	0.09 ± 0.01
		Low dose	0.09 ± 0.01	0.08 ± 0.00	0.09 ± 0.01	0.09 ± 0.01
		High dose	0.08 ± 0.01	0.08 ± 0.01	0.08 ± 0.1	0.09 ± 0.01
	QRS	Control	0.72 ± 0.01	0.73 ± 0.06	0.77 ± 0.02	0.74 ± 0.01
		Low dose	0.71 ± 0.01	0.68 ± 0.04	0.74 ± 0.02	0.70 ± 0.06
		High dose	0.72 ± 0.02	0.69 ± 0.05	0.77 ± 0.02	0.72 ± 0.05
	T	Control	0.21 ± 0.02	0.21 ± 0.2	0.26 ± 0.03	0.23 ± 0.01
		Low dose	0.25 ± 0.03	0.23 ± 0.04	0.24 ± 0.03	0.24 ± 0.02
		High dose	0.25 ± 0.03	0.24 ± 0.04	0.23 ± 0.03	0.24 ± 0.03
Duration (sec.)	*p*	Control	0.08 ± 0.01	0.09 ± 0.01	0.09 ± 0.01	0.09 ± 0.01
		Low dose	0.07 ± 0.01	0.1 ± 0.01	0.1 ± 0.01	0.1 ± 0.01
		High dose	0.08 ± 0.01	0.1 ± 0.01	0.1 ± 0.01	0.11 ± 0.01
	QRS	Control	0.1 ± 0.01	0.09 ± 0.02	0.10 ± 0.01	0.10 ± 0.01
		Low dose	0.1 ± 0.01	0.09 ± 0.01	0.09 ± 0.01	0.09 ± 0.01
		High dose	0.12 ± 0.01	0.08 ± 0.03	0.08 ± 0.01	0.09 ± 0.01
	T	Control	0.08 ± 0.01	0.08 ± 0.01	0.08 ± 0.01	0.07 ± 0.01
		Low dose	0.07 ± 0.01	0.08 ± 0.01	0.08 ± 0.01	0.08 ± 0.01
		High dose	0.08 ± 0.01	0.10 ± 0.01	0.08 ± 0.01	0.08 ± 0.01
Interval (sec.)	PR	Control	0.18 ± 0.02	0.24 ± 0.04	0.24 ± 0.03	0.23 ± 0.03
		Low dose	0.18 ± 0.01	0.21 ± 0.01	0.22 ± 0.01	0.23 ± 0.02
		High dose	0.18 ± 0.01	0.21 ± 0.01	0.22 ± 0.01	0.22 ± 0.01
	QT	Control	0.38 ± 0.04	0.45 ± 0.06	0.49 ± 0.04	0.47 ± 0.02
		Low dose	0.40 ± 0.02	0.40 ± 0.04	0.45 ± 0.02	0.45 ± 0.03
		High dose	0.39 ± 0.02	0.43 ± 0.03	0.45 ± 0.02	0.45 ± 0.01
	RR	Control	0.75 ± 0.07	0.85 ± 0.17	0.82 ± 0.18	0.78 ± 0.20
		Low dose	0.76 ± 0.12	0.76 ± 0.34	0.78 ± 0.19	0.82 ± 0.22
		High dose	0.76 ± 0.09	0.78 ± 0.49	0.74 ± 0.24	0.78 ± 0.35

**TABLE 3:** Mean (± standard deviation) serum electrolyte concentration immediately before (baseline) and up to 48 hr following two‐dose intravenous administration of low dose (2.5 mg/kg) and high dose (5 mg/kg) of furosemide, and normal saline (control) in dromedary calves (*Camelus dromedarius*)

Variables	Groups	Time points (hrs)		
		Baseline	24	48
Calcium (mg/dl)	Control	2.2 ± 0.26	2.2 ± 0.24	2.1 ± 0.29
	Low dose	2.2 ± 0.28	2.2 ± 0.29	2.1 ± 0.26
	High dose	2.2 ± 0.25	2.3 ± 0.37	2.1 ± 0.36
Phosphorus (mg/dl)	Control	2 ± 0.85	2.1 ± 0.60	1.9 ± 0.88
	Low dose	1.9 ± 0.28	2 ± 0.21	1.8 ± 0.14
	High dose	2.5 ± 0.39	2.4 ± 0.32	2.4 ± 0.45
Sodium (mEq/L)	Control	145 ± 7	147 ± 7	148 ± 4
	Low dose	149 ± 5	146 ± 7	153 ± 7
	High dose	151 ± 6	149 ± 7	149 ± 5
Potassium (mEq/L)	Control	6.1 ± 1	6.1 ± 0.9	6.1 ± 0.88
	Low dose	5.5 ± 1.2	5.8 ± 1.06	5.8 ± 1.19
	High dose	5.6 ± 0.49	6.1 ± 0.51	6.1 ± 0.91
Chloride (mEq/L)	Control	100 ± 7	96 ± 9	94 ± 8
	Low dose	91 ± 11	89 ± 11	90 ± 10
	High dose	96 ± 8	96 ± 6	97 ± 6
Magnesium (mg/dl)	Control	1.03 ± 0.21	1.05 ± 0.08	1.09 ± 0.23
	Low dose	0.84 ± 0.14	0.96 ± 0.08	1.07 ± 0.10
	High dose	1.05 ± 0.18	0.97 ± 0.21	1.03 ± 0.27

## 4 DISCUSSION

Electrolyte disturbances due to extensive administration of potent diuretics may lead to cardiac dysrhythmia. Hypokalemia is one of the common electrolyte disturbances as a side effect of diuretics (Wasfi et al., 2017). Changes in ECG after furosemide administration are largely unknown and there have been limited studies in veterinary science (Pourjafar et al., 2013). The dose of furosemide used in the current study was based on studies comparing in other ruminants (Chalmeh & Mazrouei Sebdani, 2016; Constable et al., 2017; Pourjafar et al., 2013). There were no published articles about pharmacological effects of furosemide at higher doses (such as 10 mg/kg) in dromedary calves and it may be associated with side effects and unpredictable complications. The 48‐hr time course chosen for present study was based on what is known about the pharmacokinetic characteristic of furosemide in ruminants (Ali et al., 1998; Wasfi et al., 2017). It should be noted that camelides are ‘pseudoruminants’ or ‘modified ruminants’ compared with bovine, caprine and ovine that are true ruminants. Changes in haematobiochemical parameters following furosemide have been reported in humans (Licata et al., 2003), ponies (Houpt & Perry, 2016) and horses (Raidal et al., 2014).

Electrolytes values were within normal physiological range (for this species at this age) at baseline in all study groups. This is in accordance with the finding of Samimi et al. (2019). In our study, no changes in serum electrolytes concentrations were recorded up to 48 hr. A decrease in serum levels of sodium, chloride and potassium was observed in goats 5 hr after IV furosemide (10 mg/kg) administration (Pourjafar et al., 2013). In addition, serum sodium and potassium levels were decreased after IV administration of furosemide (5 and 10 mg/kg) in high‐yielding Holstein cows (Chalmeh & Mazrouei Sebdani, 2016). In the study by Ali et al. (1998), intravenous and intramuscular administration of furosemide reduced serum electrolytes levels in adult camels. The reasons for the difference between these studies of ours and others may be due to differences in age, sex, species studied, physiological conditions and dosage of furosemide (Pourjafar et al., 2013). Wasfi et al. (2017) discussed the importance of the effect of metabolic condition in camelide on the pharmacokinetic parameters of furosemide.

In this study, all clinical variables and ECG indices at baseline were within the physiological range of camelide which was reported by Samimi et al. (2019) and Samimi et al. (2020), respectively. No significant changes in clinical variables and ECG indices were observed in any of the groups in the present study. In contrast to the current study, Pourjafar et al. (2013) observed significant changes in PR, RR and ST intervals 5 hr after IV administration of single dose of furosemide (10 mg/kg) in goats. The results of Chalmeh and Mazrouei (2016) indicated that the amplitudes of PR, RR, QT and ST intervals and also P and R amplitude after single dose of IV furosemide (5 and 10 mg/kg) were increased in high‐yielding dairy cows. Gunter‐Harrington et al. (2018) showed changes in cardiac parameters including HR and ECG indices without affecting cardiac biomarkers and ECG indices following furosemide administration in Thoroughbred horses. It can be stated that the changes in ECG indices following furosemide administration were due to changes in electrical conduction of the myocardial action potential following electrolyte imbalances (Gunter‐Harrington et al., 2018). Akita et al. (1998) confirmed the ECG changes during furosemide‐induced hypokalemia. In addition, prolonged duration of P wave and QRS complex were observed during hypokalaemia in rats (Akita et al., 1998). These results suggest that the myocardial excitabilities in the atria and ventricles may be affected by extracellular potassium level rather than by the atrioventricular conduction system in rat (Akita et al., 1998).

It should be noted that the absence of comparisons of furosemide with other diuretics as well as non‐use of higher and consecutive doses can be considered as limitations of the present study. Another limitation of this study is lack of evaluation of electrolytes levels at urinary excretion for up to 7 days.

## 5 CONCLUSION

The results obtained from the present study showed no significant changes in the clinical variables (including HR, RR, RT and ruminal motility). In addition, there were no significant differences in serum electrolytes levels and ECG indices after furosemide administration (2.5 and 5 mg/kg). Therefore, two‐dose IV injection of furosemide (2.5 and 5 mg/kg) could be administered in healthy dromedary calves under conditions with free access to fresh water. Further studies with more frequent sampling, long‐term duration, higher doses of furosemide and urine analysis are required.

## CONFLICT OF INTEREST

The authors declare that they have no competing interests.

## AUTHOR CONTRIBUTION

**Amir Saeed Samimi:** Conceptualization; Data curation; Formal analysis; Funding acquisition; Investigation; Methodology; Project administration; Resources; Writing‐review & editing. **Ali Sanjarinejad:** Software; Supervision; Validation; Visualization; Writing‐original draft.

## ETHICAL APPROVAL

All ethical considerations including animal utilization were considered cautiously. Also, the trial convention was affirmed by the animal welfare committee (which was covered IACUC approval) of the School of Veterinary Medicine, Shahid Bahonar University of Kerman, Kerman, Iran. All applicable international, national, and/or institutional guidelines for the care and use of animals were followed.
